# Effect of R119G Mutation on Human P5CR1 Dynamic Property and Enzymatic Activity

**DOI:** 10.1155/2017/4184106

**Published:** 2017-01-18

**Authors:** Linhua Li, Yujia Ye, Peng Sang, Yirui Yin, Wei Hu, Jing Wang, Chao Zhang, Deyun Li, Wen Wan, Rui Li, Longjun Li, Linling Ma, Yuehui Xie, Zhaohui Meng

**Affiliations:** ^1^Laboratory of Molecular Cardiology, Department of Cardiology, The First Affiliated Hospital of Kunming Medical University, Kunming, China; ^2^Department of Computer Science, The Faculty of Basic Medicine, Kunming Medical University, Kunming, China

## Abstract

Pyrroline-5-carboxylate reductase (P5CR1) is a universal housekeeping enzyme that catalyzes the reduction of Δ1-pyrroline-5-carboxylate (P5C) to proline with concomitant oxidation of NAD(P)H to NAD(P)^+^. The enzymatic cycle between P5C and proline is important for function in amino acid metabolism, apoptosis, and intracellular redox potential balance in mitochondria. Autosomal recessive cutis laxa (ARCL) results from a mutation in P5CR1 encoded by PYCR1. Specifically, the R119G mutation is reported to be linked to ARCL although it has not yet been characterized. We synthesized R119G P5CR1 and compared it to WT P5CR1. Foldx prediction of WT and R119G mutant P5CR1 protein stability suggests that the R119G mutation could significantly reduce protein stability. We also performed enzymatic activity assays to determine how the mutation impacts P5CR1 enzymatic function. The results of these experiments show that mutagenesis of R119 to G decreases P5CR1 catalytic efficiency for 3,4-dehydro-L-proline relative to WT. Mutagenesis and kinetic studies reveal that the activity of the mutant decreases as temperature increases from 5°C to 37°C, with almost no activity at 37°C, indicating that this mutation impairs P5CR1 function in vivo. Conversely, WT P5CR1 retains its activity after incubation at 37°C and has essentially no remaining activity at 75°C. Taken together, our experimental results indicate the R119G mutation could be an involving pathomechanism for ARCL.

## 1. Introduction

P5CR1 is encoded by the PYCR1 gene and functions as a housekeeping enzyme involved in microorganism proline synthesis [1]. The conversion of P5C to proline is catalyzed by P5CR1 in the final step of proline biosynthesis, resulting in the concomitant oxidation of NAD(P)H to NAD(P)^+^ [2]. In mammalian cells, the interconversion of proline and P5C is mechanistically impressive. By providing a metabolic shuttle of redox equivalents between the cytosol and mitochondria, P5C can be transported into cells. In cells, P5C acts as a source of oxidizing potential and its reduction to proline generates NADP^+^. Proline can be further transported into the mitochondria where in proline oxidase (POX) can regulate its conversion back to P5C, resulting in the concomitant production of NADPH [3]. Proline's metabolism-specific effect on NADP^+^/NADPH, but not NAD^+^/NADH, links proline metabolism to redox balance in mitochondria [4]. P5CR1 is also an important control point for apoptosis modulation. P5C accumulation by p53-induced POX can induce apoptosis by increasing ROS levels [5] while proline functions as a nonenzymatic antioxidant that can suppress apoptosis [6].

The importance of proline is made evident by a multitude of examples. Proline protects skin cells against photo-oxidative damage from UVA-generated singlet oxygen [7]. In the eye, a proline deficiency could affect the levels of P5C and the redox state of the lens, leading to cataracts [8]. Congenital defects in proline metabolism are known to cause neurological diseases such as seizures and significant behavioral problems (including anxiety and hallucinations) [9]. Interestingly, P5CR1 has been associated with prostate cancer [10], mammary tumors [11], and tumors of the head, neck, esophagus, and pancreas [12].

Collagen represents 80% of the extracellular matrix (ECM) and 90–95% of connective tissue [13] and can serve as a warehouse and reservoir for proline storage [14]. Autosomal recessive cutis laxa (ARCL) patients present with wrinkled, inelastic skin, joint laxity, and general connective tissue abnormalities. In ARCL patients, the inner mitochondrial membrane appears to form noncanonical, disorganized, and interrupted folds. Furthermore, the mitochondria in patient fibroblasts have a smaller diameter that indicates a role for P5CR1 in the maintenance of the mitochondrial fine structure [15]. ARCL results from an arginine to glycine mutation (R119G). This mutation appears to impair mitochondrial function [15] which could further impede catalytic function and proline metabolism in P5CR by decreasing NADH cofactor binding by the use of a molecular docking method [16, 17]. The structure of wild-type (WT) human P5CR1 has been determined by X-ray crystallography and its kinetic parameters have been described. P5CR1 is catalytically active as a homodimer, although the native enzyme exists as a decamer consisting of five homodimers [18]. The following oligomeric states have been observed in crystal form: dimer for* Nm*-P5CR1, decamer for* Sp*-P5CR1 [19], and tetramer for yeast P5CR1 [20].

Despite the elucidation of the three oligomeric states, the effect of the R119G mutation on the P5CR1 structure has not been characterized. For this reason, we synthesized the ARCL-associated R119G mutant form of P5CR1. We examined the effect of the mutation on protein stability using the Foldx algorithm. Our results show that the mutation could significantly reduce the protein's stability. We also examined how the mutation affected the enzymatic properties of P5CR1. Mutagenesis of R119 to G decreases P5CR1 catalytic efficiency. Mutagenesis and kinetic studies revealed that the activity of the mutant protein decreases as temperature increases from 5°C to 37°C with almost no activity at 37°C. This suggests that the mutation impairs P5CR1 function in vivo, and our findings demonstrate that this mutation may be linked to ARCL.

## 2. Materials and Methods

### 2.1. Protein Construction

The high-resolution X-ray crystal structure of P5CR1 (PDB ID: 2GER) [18] was obtained from Protein Data Bank (http://www.pdb.org). Arginine (R) 119 was then replaced by glycine (G) to create mutant P5CR1 using the Foldx plugin in YASARA [21].

### 2.2. Predicted Stability of Protein

The WT and mutant models were visualized using Foldx plugin in YASARA. The stability of the mutant protein structure was predicted by Foldx in YASRA software (an empirical force field-based protein design algorithm) and changes in protein stability and dynamics after mutation were evaluated [22]. The Δ*G* prediction by Foldx is the difference in free energy between the unfolded and folded state of the protein (Δ*G* =  *G*_folded_ − *G*_unfolded_). By measuring the difference of unfolding Gibbs free energy (ΔΔ*G*) between mutant and WT (ΔΔ*G* = Δ*G*_mutant_ − Δ*G*_WT_) one can calculate how much a protein mutation affects the stability. The ΔΔ*G* value predicted by Foldx is positive when the mutation is destabilizing and negative when it is stabilizing.

### 2.3. Construction of Mutant Plasmid

The mutant was constructed by PCR using the primers in Table 1. Amplification with the primers incorporated an overlap region (underlined) and resulted in the R119G site mutation (in brackets: CGC→GGC results in Arg→Gly). The complete sequence of pET28a-P5CR1 mutant was assembled by using the TransStart FastPfu Fly DNA Polymerase (TransGen Biotech, China) on WT-pET28a-P5CR1 (from our lab). The PCR reaction was a total volume of 50 *μ*L and contained 5 ng of DNA template, 0.2 *μ*M primer pairs, 250 *μ*M dNTPs, and 2.5 units of DNA polymerase. The amplification was carried out in 25 cycles: denaturation at 98°C for 20 s, primer annealing at 58°C for 30 s, and extension at 72°C for 4 min with an initial 2 min denaturation step at 98°C and a final extension step of 72°C for 5 min (Bio-Rad, USA). The PCR product was transformed into* E. coli* DH5*α*, and a transformed clone was isolated and sequenced. The R119G positive recombinant plasmid which contained the P5CR1 R119G mutation was designated as pET28a-P5CR1-R119G.

### 2.4. Expression of the R119G Mutant

The vector was transformed into* E. coli* BL21 (DE3), and the positive clone was isolated for mutant expression. The transformant was selected on LB agar plates, containing 50 *μ*g/mL kanamycin, and cells were cultured at 37°C in LB medium containing 50 *μ*g/mL kanamycin. One mM IPTG was added at mid-exponential growth phase (OD_600_ ≈ 0.6) and incubated at 30°C, 180 rpm for 6 hours. Cells were harvested by centrifugation (6080 ×g, 10°C, 15 min) (Thermo Scientific Sorvall RC 6 Plus, Germany) and resuspended in buffer A (100 mM NaH_2_PO_4_, 10 mM Tris base, pH 8.0).

### 2.5. Cell Lysis and Protein Denaturation and Renaturation

Crude cell extracts were prepared on ice by sonication in buffer A and centrifuged at 6080 ×g for 15 min at 10°C. After washing three to five times with ultrasonic cell disintegration in buffer A containing 0–2 M urea, the precipitate was denatured in buffer A containing 6 M urea with gentle shaking on ice for 2 hours. The lysate was centrifuged at 17210 ×g for 30 min at 10°C to remove the cell debris and supernatant was collected.

### 2.6. Chromatographic Purification

The supernatant was applied to a Ni^2+^-chelating column, and the column was washed with five column volumes of buffer A followed by ten column volumes of buffer A containing 6 M urea with 20 mM imidazole (pH 8.0) and eluted with buffer A containing 500 mM imidazole (pH 8.0). The eluted protein was monitored by SDS-PAGE using 12% (v/v) acrylamide. The protein was further renatured in buffer A containing 4 M urea for 8–12 hours, 3 M urea for 6–8 hours, 2 M urea for 6–8 hours, 1 M urea for 6–8 hours, and 0 M urea for 12–16 hours, respectively. The homogeneity of the purified enzyme was monitored (Gel Image System, Tanon 3500R, China) by SDS-PAGE using 12% (v/v) acrylamide gels. After concentrating the sample with an Ultrafree 10,000 NMWL filter unit (Millipore, USA) to less than 1 mL, the soluble protein was incubated with 1 mg/mL of RNaseA (Sigma, USA) and 15 U/mL of DNaseI (Takara, USA) overnight at 4°C. The Superdex-75 column (10/300 mm) was rinsed at two column volumes of distilled water at a flow rate of 0.5 mL/min and was equilibrated with buffer (20 mM Caps, pH 9.4, 0.5 M NaCl) until the UV baseline and pH were stable. The sample was injected into the column and separated using an ÄKTA purifier. The column was eluted with the equilibrated buffer and the solution was collected according to the peak of the curve. Protein concentration was determined using a protein assay kit (Sangon, China) using bovine serum albumin as a standard.

### 2.7. P5CR1 Activity Assays

The 3,4-dehydro-L-proline dehydrogenase activity of WT P5CR1 and the R119G mutant were assayed as described previously [1]. The reaction was initialized by adding mutant protein (10 *μ*L, 0.69 mg/mL) or WT protein (5 *μ*L, 0.65 mg/mL) to 200 *μ*L of reaction buffer containing 300 mM Tris-HCl (pH 9.0) (optimal condition for maximum P5CR1 activity), NAD^+^ (0.02–1.3 mM), and 3,4-dehydro-L-proline (0.02–0.44 mM). Using the mM extinction coefficient of NAD(P)H (6.22), initial rates of product formation were calculated as the increase of absorbance at 340 nm/min from the first 10 s of a 5 min recording period (UV-Visible Spectrophotometer, Thermo Evolution 260 Bio, USA). Reproducibility of all analyses was confirmed by taking each measurement at least twice. After incubation for 10 min at various temperatures (5–37°C for the mutant and 5–75°C for WT), the relative activities of P5CR1 were measured at room temperature. A sample that included both substrates without P5CR1 served as a negative control.

## 3. Results

### 3.1. Prediction of Changes in Stability due to Mutation

**Figure 1:**
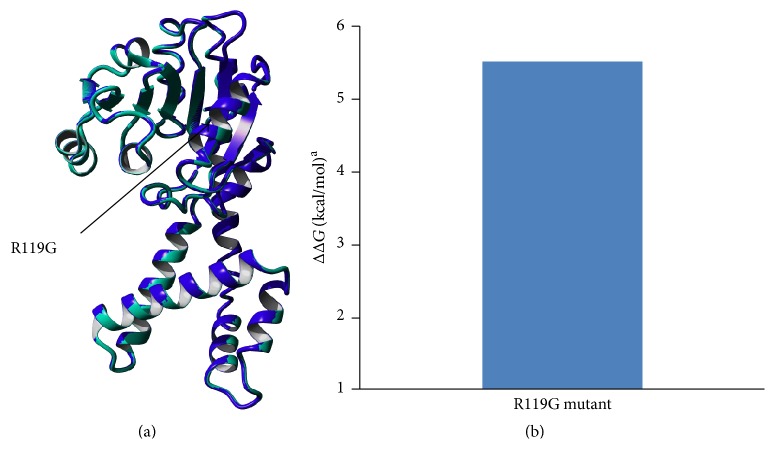
Superimposed 3D structures of WT (blue) and R119G mutant (pale green) P5CR. Changes in the protein stability upon R119G mutation predicted by Foldx. ^a^ΔΔ*G* values are changes in the protein unfolding free energy after R119G mutation. ΔΔ*G* < 0: increased stability; ΔΔ*G* > 0: decreased stability.

The produced mutant structure was compared to that of WT P5CR. The structure of the R119G mutant was nearly identical to WT (Figure 1(a) and Figure S1 in the Supplementary Material available online at https://doi.org/10.1155/2017/4184106). The effect of mutation on protein stability was predicted using Foldx (Figure 1(b)) to compare the stability of mutant P5CR1 to WT P5CR1. The predicted ΔΔ*G* value of the R119G mutant was 5.51 kcal/mol. This value implies that the site mutation could significantly reduce the protein's stability.

### 3.2. Human Mutant Is Expressed as an Inclusion Body Protein

Human mutant P5CR1 was overexpressed in* E.coli *as a fusion protein with a 6x His and T7-tag at its N-terminus under the T7 promoter (with or without IPTG induction). The majority of the mutant protein was insoluble, and there was minimal excretion into the supernatant (Fig. S2) which may imply the site mutation could affect protein folding. After denaturation the protein was purified through a Ni^2+^-chelating column, and after renaturation it was further purified by size exclusion chromatography (SEC). The P5CR1 recovered was associated with a minimal amount of nucleic acids that could then be digested by incubation with RNaseA and DNaseI.

### 3.3. Human Mutant Forms a Dimer

**Figure 2:**
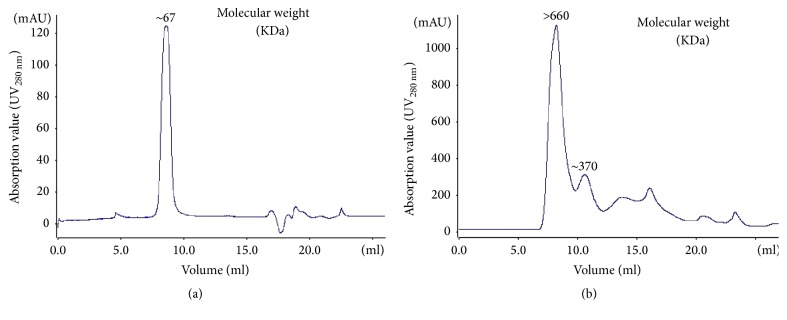
(a) Human R119G mutant shows a peak (~67 KDa) by Superdex-75 column. (b) Human WT shows peak 1 (>660 kDa) and peak 2 (~370 kDa) from Superdex-200 column.

**Figure 3:**
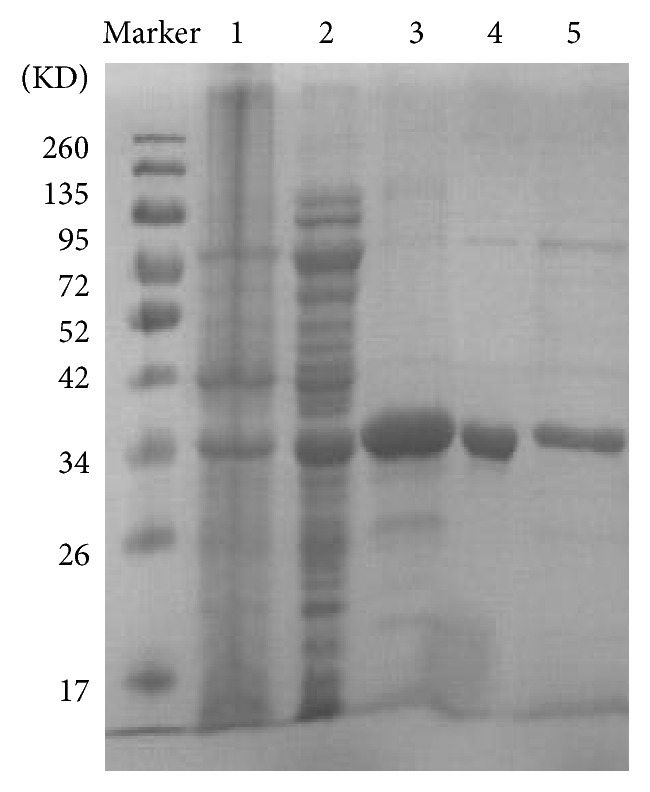
Purification analysis of R119G mutant by 12% SDS-PAGE. Marker, molecular mass markers. 1, before induction. 2, after induction. 3, mutant purified by Ni^2+^-chelating column. 4, mutant purified by SEC. 5, WT purified by SEC.

The purified mutant protein presented with a peak at ~67 kDa in 8–10 mL (Figure 2(a)). This indicates that the R119G mutant could form a bipolymer by SEC as its monomer molecular weight is 33.3 kDa. The WT presented with two peaks: peak 1 at >660 kDa and peak 2 at ~370 kDa (Figure 2(b)). The target protein was determined by Coomassie Brilliant Blue R-250 staining (Figure 3) and farther utilized for enzymatic activity assays.

### 3.4. Enzymatic Activity Assay of Human WT and Mutant P5CR1

We analyzed P5CR1 enzymatic activity with a proline dehydrogenase assay (P5CR1 inverse reaction). The enzyme was assayed by dehydrogenation of the 3,4-dehydro-L-proline. The kinetic parameters for WT and mutant P5CR1 were determined (Table 2). The WT *V*_max_ and *K*_*m*_ values using proline as the variable substrate with fixed NAD^+^ are 0.27 mM/min and 0.20 mM, respectively. The R119G mutant *V*_max_ and *K*_*m*_ values using proline as the variable substrate with fixed NAD^+^ are 0.12 mM/min and 1.28 mM. These results suggest a decrease in substrate affinity for the R119G mutant when compared to WT. When using NAD^+^ as the variable substrate with fixed 3,4-dehydro-L-proline for WT, the *V*_max_ and *K*_*m*_ values are 0.21 mM/min and 0.09 mM, respectively. The R119G mutant under the same conditions has *V*_max_ and *K*_*m*_ value of 0.04 mM/min and 0.59 mM, respectively. These also suggest a decrease in affinity of the mutant for its cofactor when compared to WT.

Overall our results demonstrate that catalytic activity is severely impaired in the mutant. Using 3,4-dehydro-L-proline as the variable substrate, the *k*_cat_ values for the WT and mutant were 10.23 s^−1^ and 2.13 s^−1^, respectively. The catalytic efficiency was measured with 3,4-dehydro-L-proline and was roughly 30 times lower for the mutant (1664 s^-1 ^M^−1^) when compared to WT (1150 s^-1 ^M^−1^). When NAD^+^ was used as the variable substrate, the *k*_cat_ values were 11 times lower in the mutant compared to WT (0.71 s^−1^ and 7.95 s^−1^, resp.). The catalytic efficiencies showed a 73-fold difference between the WT and mutant enzymes for NAD^+^ with 88333 s^−1^ M^−1^ for the WT and 1203 s^−1^ M^−1^ for the mutant.

**Figure 4:**
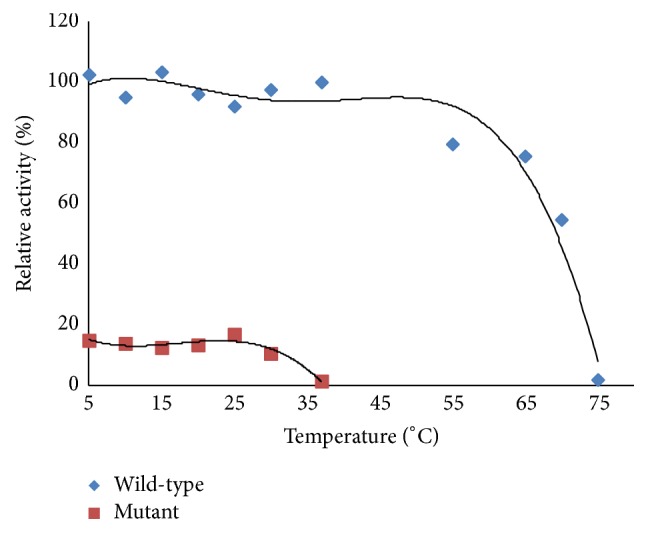
Comparison of thermal effects between the mutant and WT. After enzymes incubated at various temperatures (5–37°C for the R119G mutant and 5–75°C for WT) for 10 min, the relative activities of WT and mutant were measured at room temperature.

The activity of the R119G mutant decreased as the temperature increased from 5°C to 37°C. At 37°C, there was essentially no activity. In contrast, WT P5CR1 retained its activity after incubation at 37°C and had almost no activity at 75°C. The thermal effects of the mutation are shown in Figure 4.

## 4. Discussion

Our study characterized the differences between the R119G mutant and WT form of P5CR1. The mutant protein was primarily inclusion body while most of the WT protein was soluble. In comparison to the WT form that is a decamer [23], the mutant protein could form a dimer. Arginine 119 is located on the *β*6 strand of the A domain of P5CR1 [18], and the substitution of a much smaller amino acid (such as glycine) at this site could likely affect the protein's characteristics. When glutamic acid 221 is mutated to glycine or the N-terminus is truncated, P5CR1 protein results in bodies in* E. coli* [18]. When taken together, this evidence suggests that the R119G mutation could influence the protein folding of P5CR1.

Activity of the R119G mutant decreased as temperature increased from 5°C to 37°C, and the mutant enzyme almost completely lost its activity at 37°C. Alternatively, WT P5CR1 still had activity at 37°C and almost completely lost its activity at 75°C, which suggests the mutant to be thermolabile. The structure of the R119G mutant was nearly identical to WT. Our model of mutant protein structure stability (through Foldx in YASRA software) showed that the protein was destabilized by the R119G mutation. Mutagenesis of R119 to G decreased P5CR1's catalytic efficiency 30-fold for 3,4-dehydro-L-proline and 14-fold for cofactors. Catalytic activity was severely impaired in the mutant. R119 has been shown to take part in forming the binding site of the protein, and mutation of this site could destabilize the binding of NADH via hydrogen bonds [18]. Additionally, the crystal structure of P5CR1 demonstrates a decameric architecture with five homodimer subunits and ten catalytic sites arranged around a peripheral circular groove [18]. Therefore, we hypothesized that decreased activity of the mutant could be due to changes in the number of catalytic sites. The binding site consists of two main parts: the hydrophobic wall and the charged center. The hydrophobic wall is formed by Phe158(A), Arg200(H), and Arg204(H). The charged pocket, which stabilizes NADH with hydrogen bonds, is formed by residues in the *α*7,  *α*10, and *α*11 helices, the *β*6–*β*8 strands, and the loops/turns among them. Arg129(A) forms the ceiling of this pocket; His219(A) and Glu130(A), Ser154(A), Gly157(A), and Gln208(H) form the right-hand and left-hand wall of this pocket [18]. This pocket contributes significantly to cofactor binding as these residues contact the bound cofactor largely via hydrogen bonds. Molecular dynamics (MD) simulation predictions reveal that the cofactor binding affinity and catalytic efficiency of P5CR1 are decreased through either the decrease in structural stability of P5CR or the reduction of P5CR1's binding affinity toward its essential cofactor NAD [16]. The change in activity for the R119G mutant predicted by the modeling simulation is highly consistent with our experimental data suggesting the activity of mutant is likely decreased relative to WT. Additionally, the arginine at this position is located on the *β*6 strand of the A domain, the residues of which are implicated in the formation of the binding sites' charged pocket [18]. Mutation of R119 to G could influence the charged pocket of the binding sites. Our study demonstrates that the change of activity in the R119G mutant could be related to ARCL. The affinity for substrates is decreased with the mutant enzyme (*K*_*m*_ = 1.28 mM) when compared to WT (*K*_*m*_ = 0.20 mM). Moreover, the affinity for the NAD cofactor is decreased in mutant enzyme (*K*_*m*_ = 0.59 mM) when compared to WT (*K*_*m*_ = 0.09 mM). Results from molecular docking reveal that the NAD cofactor binding affinity of P5CR1 is reduced in the mutant [16]. The affinity of WT P5CR1 for NAD^+^ is 20-fold higher than its affinity for NADP^+^, while the affinity of the E221A mutant for NAD^+^ is only twofold higher than that for NADP^+^ [18]. Arginine 119 is located on the *β*6 strand of the A domain, the residues of which compose the charged pocket of the binding sites [18] which may indicate that the mutation significantly alters P5CR1's Km.

**Figure 5:**
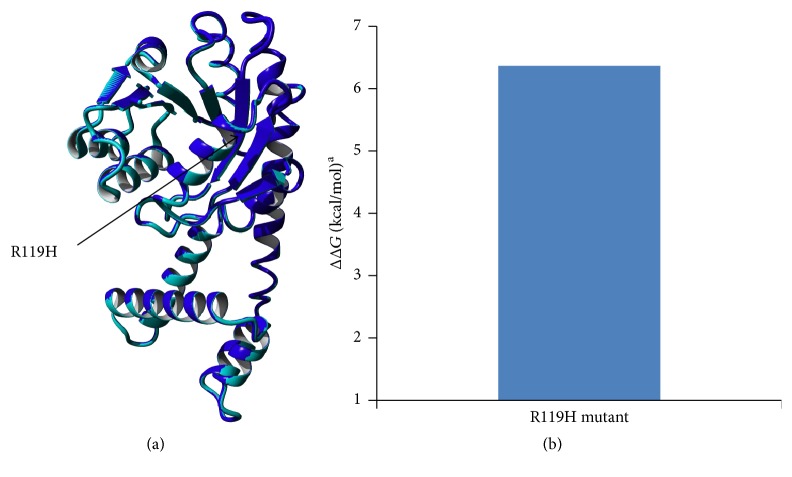
(a) Superimposed 3D structures of WT (blue) and R119H mutant (pale green) P5CR. (b) Changes in the protein stability upon R119H mutation predicted by Foldx. ^a^ΔΔ*G* values are changes in the protein unfolding free energy after mutation. ΔΔ*G* < 0: increased stability; ΔΔ*G* > 0: decreased stability.

**Figure 6:**
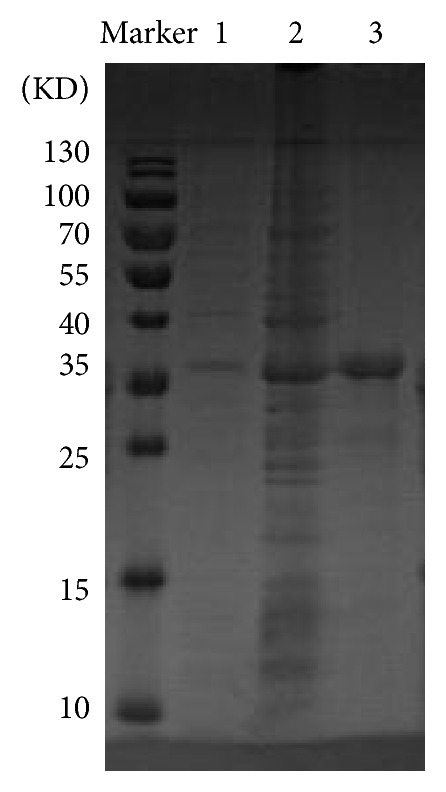
Purification analysis of R119H mutant by 12% SDS-PAGE. Marker, molecular mass markers. 1, before induction. 2, after induction. 3, mutant purified by Ni^2+^-chelating column.

**Figure 7:**
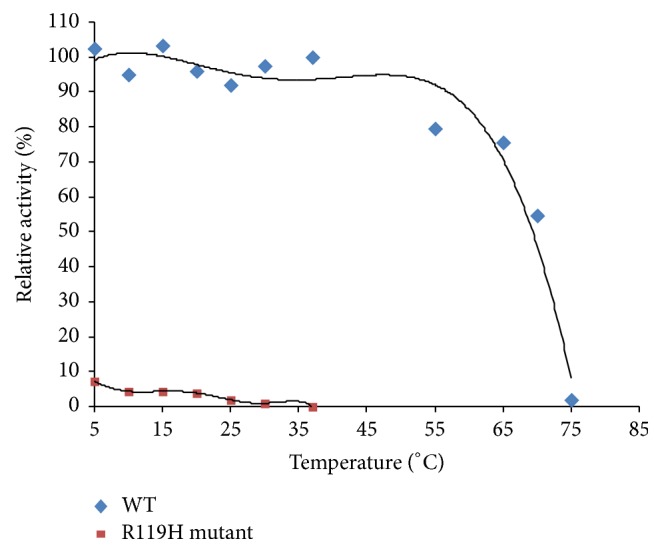
Comparison of thermal effects between the mutant and WT. After enzymes incubated at various temperatures (5–37°C for the R119H mutant and 5–75°C for WT) for 10 min, the relative activities of WT and mutant were measured at room temperature.

The structure of the R119H mutant was nearly unchanged compared to of that the WT (Fig. S1, Figure 5(a)), and it showed ΔΔ*G* > 0 (Figure 5(b)) which suggests its stability is decreased. We successfully constructed the R119H mutant plasmid by the previous method, and most of the expressed protein was insoluble as primarily inclusion bodies (Fig. S3). The R119H mutant protein was purified with a Ni^2+^-chelating column (Figure 6), and the protein could form a dimer by SEC (Fig. S4). The activity of the R119H mutant was consistent with that of the R119G mutant which strongly implies that activity will decrease as the temperature increases from 5°C to 37°C with no remaining activity at 37°C (Figure 7). Taken together, mutagenesis of R119 to G or H could impair P5CR1 function in vivo. In another genetic disorder that is characterized by microcephaly and hypomyelination (the absence of lax and wrinkled skin), a mutation has been identified at residue Arg119 in PYCR2 which corresponds to the same mutated region in PYCR1 in affected individuals. Loss of function in P5CR2 leads to decreased mitochondrial membrane potential and increased susceptibility to apoptosis under oxidative stress. Hypomyelination is a distinguished characteristic in ARCL [24].

Our study demonstrates that the two site mutations, R119G and R119H, could influence the function of the P5CR1 protein, which could be a pathomechanism for ARCL. Our results highlight the importance of the “charged pocket” in P5CR1 function which could provide a new treatment strategy for ARCL in the future.

## Supplementary Material

The 3D structures of the R119G and R119H mutants were nearly identical to WT. (A) WT; (B) R119G; mutagenesis of Arginine 119 to Glycine (C) R119H; mutagenesis of Arginine 119 to Histidine

## Figures and Tables

**Table 1:** The R119G mutant was constructed using PCR by the following pair of primers.

Mutant	Primers
R119G	5′-CCAGCCCCCAGGGTCATC(G)GCTGCATGACCA-3′
	5′-(C)GATGACCCTGGGGGCTGGCCGAAACGCTGAC-3

**Table 2:** Kinetic parameters for WT and mutant enzymes using 3,4-dehydro-L-proline as substrate and NAD^+^ as cofactor.

	*k*_cat_ (s^−1^)	*K*_*m*_ (mM)	*V*_max_ (mM/min)	*k*_cat_/*K*_*m*_ (s^−1^M^−1^)
	3,4-Dehydro-L-proline as the variable substrate^a^			
WT	10.23	0.20	0.27	51150
Mutant	2.13	1.28	0.12	1664
	NAD^+^ as the variable substrate^b^			
WT	7.95	0.09	0.21	88333
Mutant	0.71	0.59	0.04	1203

^a^[NAD^+^] was fixed at 1.0 mM.

^b^[3,4-Dehydro-L-proline] was fixed at 352 *μ*M.
