# Supplementary material for: Effect of R119G Mutation on Human P5CR1 Dynamic Property and Enzymatic Activity
Source: Biomed Res Int. 2017 Jan 18;2017:4184106. doi: 10.1155/2017/4184106 (PMC5286483; doi:10.1155/2017/4184106)
Supplement: Supplementary file 1 — The 3D structures of the R119G and R119H mutants were nearly identical to WT. (A) WT; (B) R119G; mutagenesis of Arginine 119 to Glycine (C) R119H; mutagenesis of Arginine 119 to Histidine [file 4184106.f1.pdf]

Supplementary Information

Table S1 The R119H mutant was constructed using PCR by the following pair of primers.

Mutant	Primers
R119H	5'-CAGCCCCCAGGGTCATCC(A)CTGCATGACCAAC-3'<br>5'- (T)GGATGACCCTGGGGGCTGGCCGAAACGCTGAC-3'

(in brackets, CGC → CAC results in Arg → His).

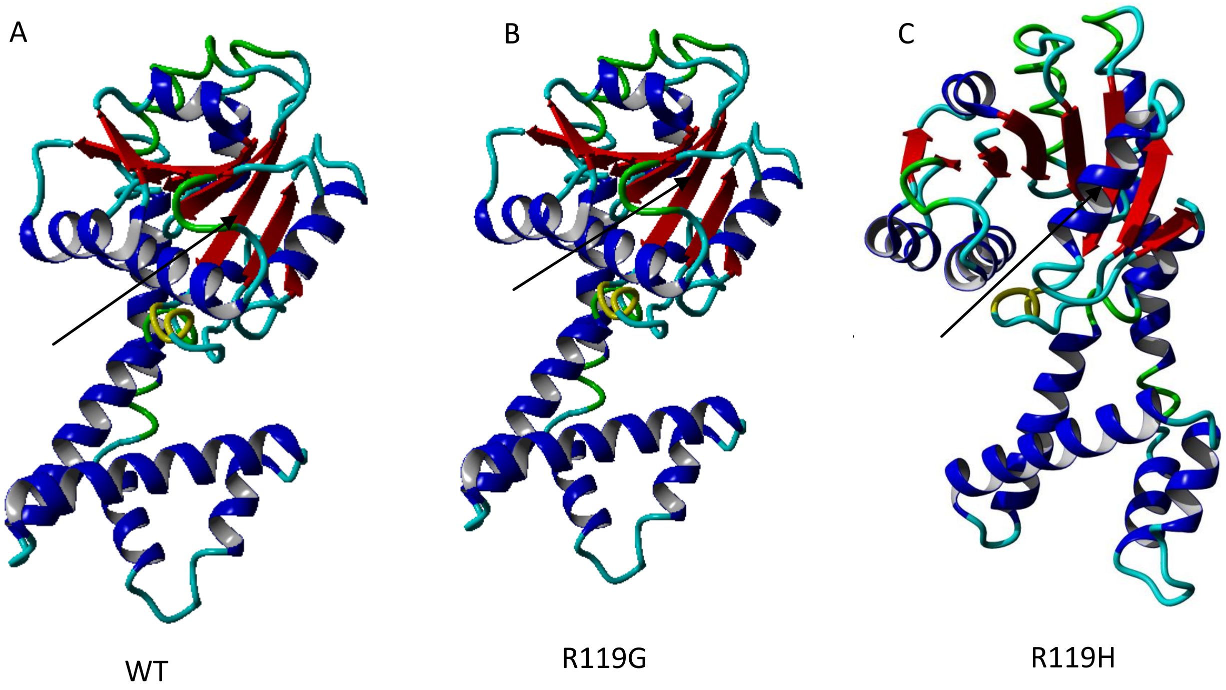

Fig.S1 3D structures of WT and mutants P5CR. (A) WT; (B) R119G; (C) R119H

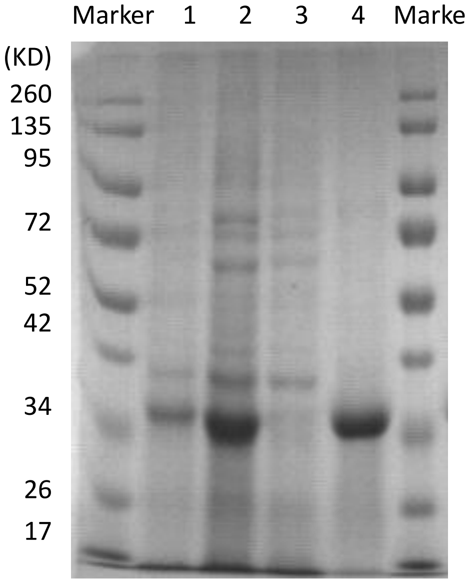

Fig.S2 Expression analysis of R119G mutant by 12% SDS-PAGE; Marker, molecular mass mark.1,before induction.2, after induction. 3, supernatant. 4, precipitate.

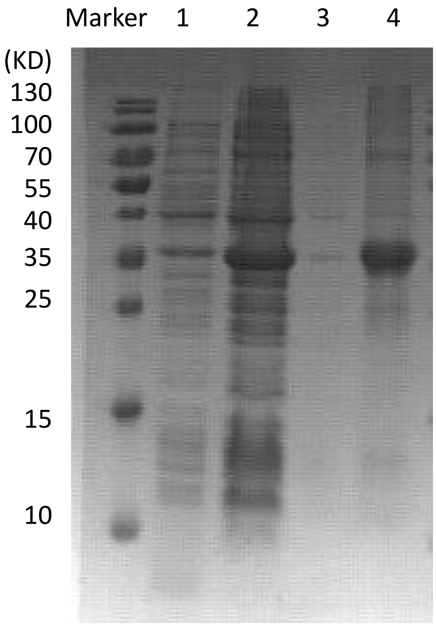

Fig.S3 Expression analysis of R119H mutant by 12% SDS-PAGE; Marker, molecular mass mark. 1, before induction.2, after induction. 3, supernatant. 4, precipitate.

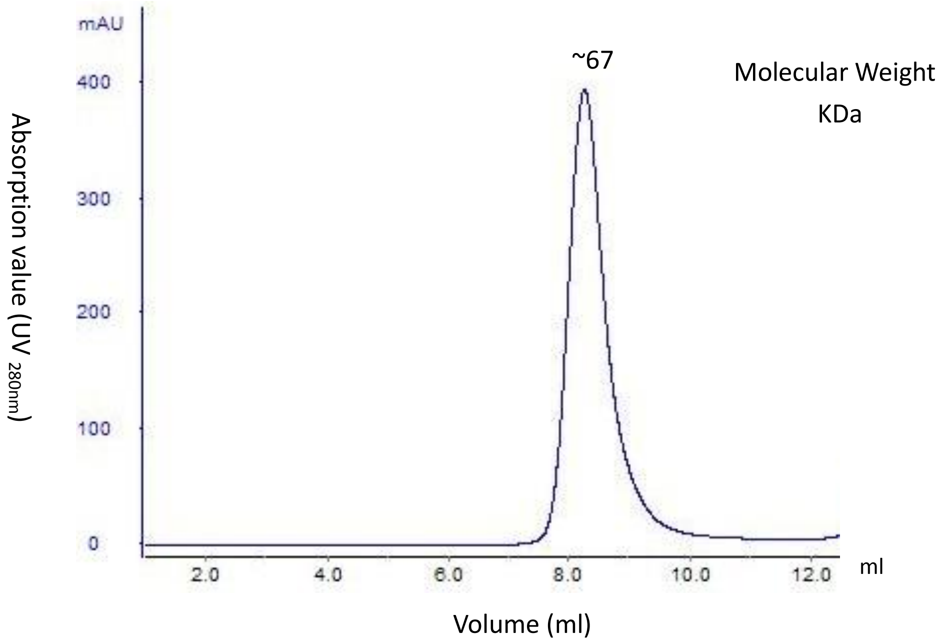

Fig. S4. Human R119H mutant shows a peak (~67KDa) by Superdex-75 column.
